# Quantitative accuracy of preclinical *in ovo* PET/MRI: influence of attenuation and quantification methods

**DOI:** 10.1186/s40658-024-00714-3

**Published:** 2025-01-21

**Authors:** Theresa Balber, Katarína Benčurová, Manuela Mayrhofer, Joachim Friske, Martin Haas, Claudia Kuntner, Thomas H. Helbich, Marcus Hacker, Markus Mitterhauser, Ivo Rausch

**Affiliations:** 1 Joint Applied Medicinal Radiochemistry Facility, University of Vienna, Medical University of Vienna, Vienna, Austria; 2 Division of Nuclear Medicine, Department of Biomedical Imaging and Image-Guided Therapy, Medical University of Vienna, Vienna, Austria; 3 School of Medical Engineering and Applied Social Sciences, University of Applied Sciences Upper Austria, Linz, Austria; 4 Division of Molecular and Structural Preclinical Imaging, Department of Biomedical Imaging and Image-Guided Therapy, Medical University of Vienna, Vienna, Austria; 5 Bruker BioSpin GmbH & Co. KG, Ettlingen, Germany; 6 Department of Biomedical Imaging and Image-Guided Therapy, Medical University of Vienna, Vienna, Austria; 7 Medical Imaging Cluster, Medical University of Vienna, Vienna, Austria; 8 Department for Inorganic Chemistry, Faculty of Chemistry, University of Vienna, Währinger Straße 42, Vienna, 1090 Austria; 9 QIMP Team, Center for Medical Physics and Biomedical Engineering, Medical University of Vienna, Vienna, Austria

**Keywords:** HET-CAM model, In ovo imaging, PET/MRI, Attenuation correction

## Abstract

**Aim:**

The combination of positron emission tomography (PET) and magnetic resonance imaging (MRI) provides an innovation leap in the use of fertilized chicken eggs (*in ovo* model) in preclinical imaging as PET/MRI enables the investigation of the chick embryonal organ-specific distribution of PET-tracers. However, hybrid PET/MRI inheres technical challenges in quantitative *in ovo* PET such as attenuation correction (AC) for the object as well as for additional hardware parts present in the PET field-of-view, which potentially contribute to quantification biases in the PET images if not accounted for. This study aimed to investigate the influence of the different sources of attenuation on *in ovo* PET/MRI and assess the accuracy of MR-based AC for *in ovo* experiments.

**Method:**

An in-house made chicken egg phantom was used to investigate the magnitude of self-attenuation and the influence of the MRI hardware on the PET signal. The phantom was placed in a preclinical PET/MRI system and PET acquisitions were performed without, and after subsequently adding the different hardware parts to the setup. Reconstructions were performed without any AC for the different setups and with subsequently incorporating the hardware parts into the AC. In addition, *in ovo* imaging was performed using [^18^F]FDG and [^68^Ga]Ga-Pentixafor, and PET data was reconstructed with the different AC combinations. Quantitative accuracy was assessed for the phantom and the *in ovo* measurements.

**Results:**

In general, not accounting for the self-attenuation of the egg and the hardware parts caused an underestimation of the PET signal of around 49% within the egg. Accounting for all sources of attenuation allowed a proper quantification with global offsets of 2% from the true activity. Quantification based on % injected dose per cc (%ID/cc) was similar for the *in ovo* measurements, regardless of whether hardware parts were included in AC or not, when the injected activity was extracted from the PET images. However, substantial quantification biases were found when the self-attenuation of the egg was not taken into account.

**Conclusion:**

Self-attenuation of the egg and PET signal attenuation within the hardware parts of the MRI substantially influence quantitative accuracy in *in ovo* measurements. However, when compensating for the self-attenuation of the egg by a respective AC, a reliable quantification using %ID/cc can be performed even if not accounting for the attenuation of the hardware parts.

## Introduction

The chick embryo and its chorioallantoic membrane (CAM) have been used in biomedical research for more than a century and have led to breakthrough discoveries in cardiovascular research, tumor biology, and embryonic development [1]. For the latter, structural imaging modalities such as computed tomography (CT) and magnetic resonance imaging (MRI) have been used to noninvasively monitor anatomical and morphological aspects of the developing chick embryo [2–5]. In the last decade, the use of fertilized chicken eggs as an alternative model to rodents has also become of interest to the radiopharmaceutical community for ethical and economic reasons. However, *in ovo* (chick embryos growing inside the natural eggshell) imaging using functional modalities such as positron emission tomography (PET) is still in its infancy. So far, *in ovo* PET imaging has been applied with a strong focus on imaging xenografts that have been previously inoculated onto the CAM [6–11]. Little attention has been paid to embryonal organ uptake, presumably because chicken embryos are comparatively small and the resolution of the available combined structural imaging techniques (CT) with PET was previously insufficient to allow for appropriate delineation of embryonal organs.

The recently introduced combination of µPET/MRI provides an innovation leap in preclinical imaging [12]. High magnetic field MRI enables high resolution of small soft tissue structures and, therefore, the investigation of the chick embryonal organ-specific distribution of PET-tracers, when correctly co-registered and quantified. Until now, *in ovo* PET/MRI has been mainly measured sequentially on separate devices [9, 13]. While this is a viable approach in case a dedicated PET/MRI system is not available, the use of a hybrid PET/MRI system allows for simultaneous acquisition of both signals leading to reduced scanning times and anesthesia for the embryo [14]. Furthermore, in simultaneous acquisitions inaccuracies in image registration due to embryonal movement within the egg are mitigated.

However, hybrid PET/MRI inheres technical challenges that need to be considered when performing quantitative *in ovo* PET. As for all PET examinations, attenuation correction (AC) for the object under investigation as well as the used hardware parts (e.g. MRI coils) has to be performed. This can be done similarly to clinical PET/MR systems using specific MRI sequences and attenuation templates for rigid hardware parts [15]. However, most of the methods are tailored to human subjects and clinical PET/MRI, whereas MR-based AC is only sparsely evaluated in preclinical systems [16, 17]. Moreover, no information on the quantitative accuracy of *in ovo* PET/MRI is available. Quantification of tracer concentration in terms of percent injected dose per gram tissue (%ID/g) and standardized uptake values (SUV) or the use of kinetic modeling to quantify the kinetics of the radiotracer are widely used techniques in preclinical imaging [18]. Yet, such approaches need to be accurate, specifically, if used for studies comparing different groups or when results should be comparable to results from other groups or used in multicenter studies [19]. Therefore, we aimed to close this knowledge gap and to investigate the influence of the different sources of attenuation on *in ovo* PET/MRI measurements. Further, the accuracy of MR-based AC for *in ovo* experiments was assessed and recommendations on practical quantification strategies are given.

## Materials and methods

All measurements were performed on a preclinical 9.4T MRI system (BioSpec^®^ 94/30 USR with 9.4 Tesla, Bruker Biospin GmbH & Co. KG, Germany) coupled with a PET insert (PET insert Si 198, Bruker Biospin GmbH & Co. KG, Germany) [20] in the preclinical imaging laboratory (PIL) of the Medical University of Vienna. The scanner has a field-of-view (FoV) of 150 mm (axial) and 80 mm (radial). The *in ovo* methodology used in this study does not represent animal experimentation according to Austrian law, as no living vertebrates are used, born or hatched (§ 1 Abs.1 TVG 2012, § 2 Z 1 lit. b TVG 2012). Furthermore, this model is not recognized as a protected species until embryonal development day (EDD)14 according to European law (directive 2010/63/EU) and experimentation can be performed up to EDD14 without ethical approval. Nevertheless, all procedures involving chicken embryos were performed with the utmost care in anesthesia and euthanasia by exclusively trained personnel and under veterinary supervision.

### Phantom measurements

**Fig. 1:**
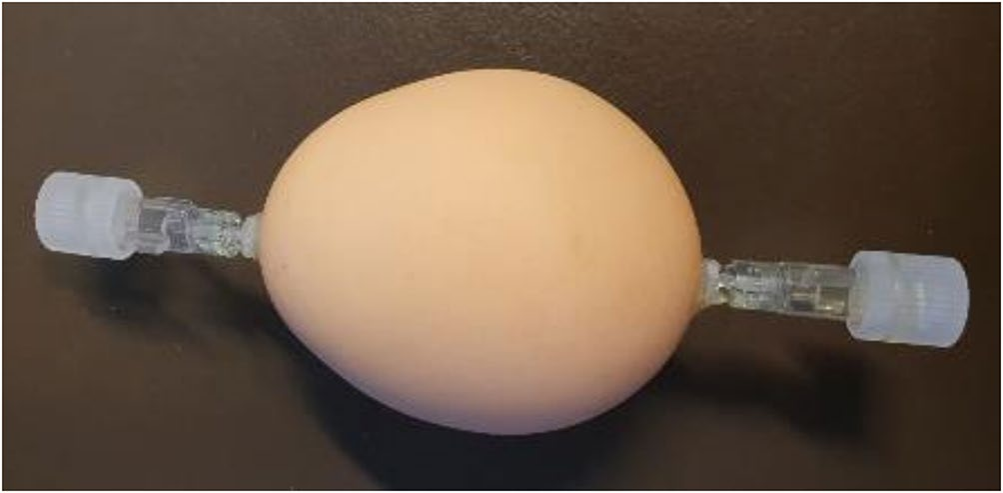
Chicken egg phantom used for the assessment of self-attenuation

To investigate the magnitude of self-attenuation and the influence of the different hardware parts on the PET signal, phantom measurements were performed. Therefore, a chicken egg phantom was built by inserting two small holes along the long axis of a standard hen’s egg. The albumin and yolk were extracted and the egg was cleaned. Then two sealable syringe connectors from an infusion line (RoweMed, Germany) were tightly connected to the two holes using a UV-curable glue (LED Light Booster, UHU GmbH, Germany) to allow filling of the eggs with an aqueous radioactive solution (Fig. 1).

The egg phantom was subsequently filled with a physiological saline solution containing [^18^F]FDG (later referred to as actual activity concentration) and positioned with the long axis of the egg aligned with the z-axis of the MRI in the center of FOV of the PET/MRI. Measurements were done using five different hardware configurations (HC):

#### HC-1

the phantom was positioned on a Styrofoam holder (density ~ 0.01 g/cc) with no additional hardware parts to assess the attenuation of the egg only.

#### HC-2

the phantom was placed on the animal bed (Model nr. T12560, Bruker Biospin GmbH & Co. KG, Germany), thus adding the contribution of the bed to the attenuation.

#### HC-3 to HC-5

the phantom was placed on the animal bed as for HC-2. In addition, three different cylindrical MR coils (Bruker Biospin GmbH & Co. KG, Germany) were inserted into the PET insert. HC-3: Model nr.: T9361V3 / RF RES 400 1 H 112/072 QSN TR AD, 72 mm inner diameter; HC-4: Model nr.: T20204V3 / RF RES 400 1 H 112/072 QSN TR AD PO, 72 mm inner diameter, coil optimized for PET measurements; HC-5: Model nr.: T20202V3 / RF RES 400 1 H 112/086 QSN TR AD PO, 86 mm inner diameter, coil optimized for PET measurements. All coils are rigid objects which are reproducibly placed at a specific position within the PET insert. The term “optimized for PET measurements” refers to a reduced material content within the PET FOV to minimize the absorption of the PET signal.

For each setup, three consecutive PET acquisitions of 5 min each were performed using a 30% (358 keV – 664 keV) energy and 7 ns coincidence timing window. The activity concentration in the phantom at the start of each measurement is given in Table 1.

For HC-4 and HC-5 additional MR imaging was performed for attenuation correction using a Fast Low Angle Shot (FLASH) sequence (TR = 12 ms, TE = 3.6 ms) with a matrix size of 180 × 180 × 300 with an isotropic voxel size of 0.5 × 0.5 × 0.5 mm³.

### PET image reconstruction and attenuation correction for phantom scans

All PET data was reconstructed using the standard Maximum Likelihood Expectation Maximization Algorithm (MLEM) available on the PET/MR system using 12 iterations. A matrix size of 90 × 90 × 150 was used resulting in a pixel size of 1 × 1 × 1 mm³. Standard corrections for normalization, deadtime, randoms, intra-frame decay and scatter were included in all reconstructions. For this system, normalization is a direct normalization using a ^68^Ge/^68^Ga cylinder, deadtime correction is based on deadtime measurements over a range of activities performed by the vendor, randoms correction is based on a randoms estimated using the singles rate and the scatter correction is based on the dual-energy window method.

First, all acquisitions were reconstructed without any AC. For HC-4 and HC-5 additional reconstructions were performed using different AC configurations:

#### AC-1

including only the AC map of the egg. This AC map is the standard AC method implemented in the system and is based on a segmentation of the acquired MRI into air and tissue. The tissue compartment was subsequently assigned a linear attenuation coefficient of 0.1023 cm^− 1^.

#### AC-2

including the AC map of the egg and the animal bed. The mouse bed AC template was based on a CT measurement of the bed, which was scaled to attenuation coefficients of 511 keV using bilinear scaling [21]. The template was then implemented into the PET/MR software (Paravision 360 V3.2 software, Bruker Biospin GmbH & Co. KG, Germany) to allow a retrospective addition to the AC map of the egg.

#### AC-3

including the AC map of the egg, the animal bed and the respective MR coil. The MR coils were added similar as the animal bed using AC templates. The AC templates of the PET optimized coils (HC-4 and HC-5) are based on CAD drawings from the vendor and contain known attenuation coefficients of the used materials.

### Phantom data evaluation

For all acquisitions and reconstructions, the activity concentrations in a volume-of-interest (VOI) containing the whole liquid compartment of the egg phantom, and from two spherical VOIs of 1 cm diameter each, placed in the center of the egg and near the eggshell were extracted and compared to the actual activity concentration. Further, for all reconstructions, the three consecutive acquisitions were filtered with a 2 mm FWHM Gaussian filter and combined in one average image. These combined and filtered image data sets were used to create line profiles normalized to the actual activity concentration through the short axis of the egg and percent difference images. Data processing was done using MATLAB (v. R2020b, MathWorks, USA) and the software Amide (v. 1.0.4; [22])

### In ovo PET/MR measurements

In preclinical PET, one of the most commonly used semi-quantitative parameters is the ratio of the activity in a tissue (kBq/cc) divided by the decay-corrected activity injected into the animal (kBq), yielding the percent injected dose per cubic centimeter tissue (%ID/cc) [18]. To assess the influence of attenuation on this standard quantification metrics, 10 *in ovo* PET/MRI measurements were included in this evaluation. To this, 5 datasets from the development phase of the methodology (using [^18^F]FDG, dataset 1) and 5 datasets from a subsequent study (using [^68^Ga]Ga-Pentixafor, dataset 2) were selected. The rationale for including datasets from the development phase was to also identify potential experimental and methodological pitfalls affecting quantification.

For both groups, specific pathogen-free, fertilized chicken eggs (quality: Premium Plus, Charles River Laboratories, Research Models and Services, Germany GmbH, Sulzfeld, Germany) were incubated under standardized conditions (37 °C, 50–60% humidity) in the horizontal position using a dedicated automated incubator (Wiltec Wildanger Technik GmbH, Eschweiler, Germany). The eggs were punctured on day 3 of incubation for ventilation. This window was enlarged on the following days to allow tumor cell inoculation (HT29 or HCT116) onto the CAM on embryonal development day 9 following an established protocol [14]. Imaging was performed between day 14 and 18 of embryonal development. To this, 4.89 ± 2.26 MBq [^18^F]FDG (dataset 1) or 10.32 ± 8.08 MBq (corresponding to 1.80 ± 0.80 nmol) [^68^Ga]Ga-Pentixafor (dataset 2) were injected into a CAM vessel using self-assembled catheters connected to 30G needles [14]. Eggs were anesthetized using 3% isoflurane in 2 L air/min and placed in the center of the PET/MR in HC-5 (see *Phantom measurements*). For each egg, a 15 min PET acquisition was started 60 min post injection of the tracer using the same energy and coincidence timing window settings as for the phantom acquisitions. MR acquisitions included the FLASH sequence for attenuation correction similar as for the phantom measurements and additional anatomical TurboRARE sequence including the whole egg and specifically the CAM xenograft. 2% isoflurane in 2 L air/min was used to maintain anesthesia throughout the imaging procedure.

PET data reconstruction was performed using no AC (NO-AC) and using the different AC configurations as for the phantom measurements, with a MLEM algorithm applying 16 iterations. A matrix size of 180 × 180 × 300 was used resulting in a pixel size of 0.5 × 0.5 × 0.5 mm³. All standard corrections were applied as for the phantom measurements.

### In ovo data evaluation

**Fig. 2:**
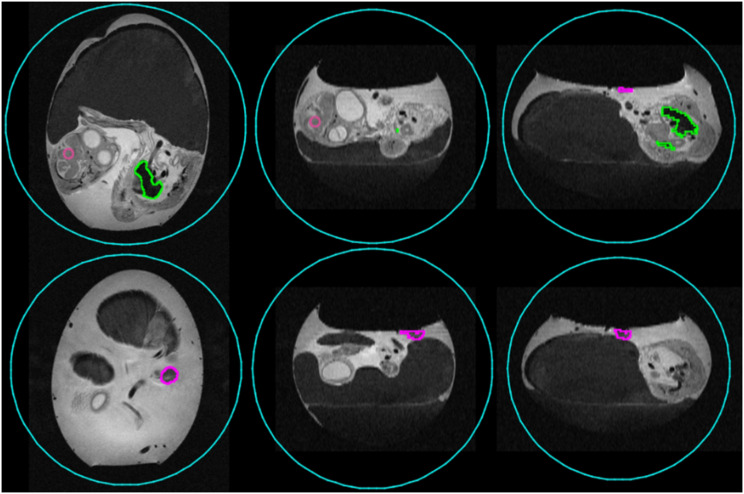
Example for VOI positioning. VOIs for liver (green) and xenograft (purple) were manually drawn based on the anatomical information from the T2-weighted TurboRARE scans (axial). A sphere (1 mm radius, rosa) was positioned in the center of the brain with the help of coregistered PET data as to mitigate spill-in effects. A sphere with a radius of 30 mm was placed around the whole egg to assess the total activity

To evaluate quantitative accuracy of the *in ovo* experiments, activity concentrations (kBq/cc) were extracted for the brain, the liver, and the xenograft using dedicated image analysis software (Pmod version 3.807, PMOD Technologies, Zürich, Switzerland). VOIs for xenograft and liver were manually delineated based on the T2-weighted TurboRARE MR sequences. To mitigate spill-in effects, in particular for the Ga-68 tracer, a spherical VOI with 1.2 mm in radius was placed in the middle of the brain to assess brain uptake. To determine the total activity within the egg, a spherical VOI (30 mm radius) was placed around the whole egg. An example for the VOI placement can found in Fig. 2.

For the whole egg, the total activity determined from the images was compared to the injected activity as determined with the dose calibrator. To quantify the tissue uptake, % ID/cc was calculated for all AC configurations by dividing the activity concentration in the respective tissue with the total activity determined from the PET images. For AC-3, %ID/cc was additionally calculated by dividing the activity concentration in the tissue by the injected activity determined by the dose calibrator (by measuring the initial and residual activity in the syringe).

## Results

### Phantom evaluation

**Fig. 3:**
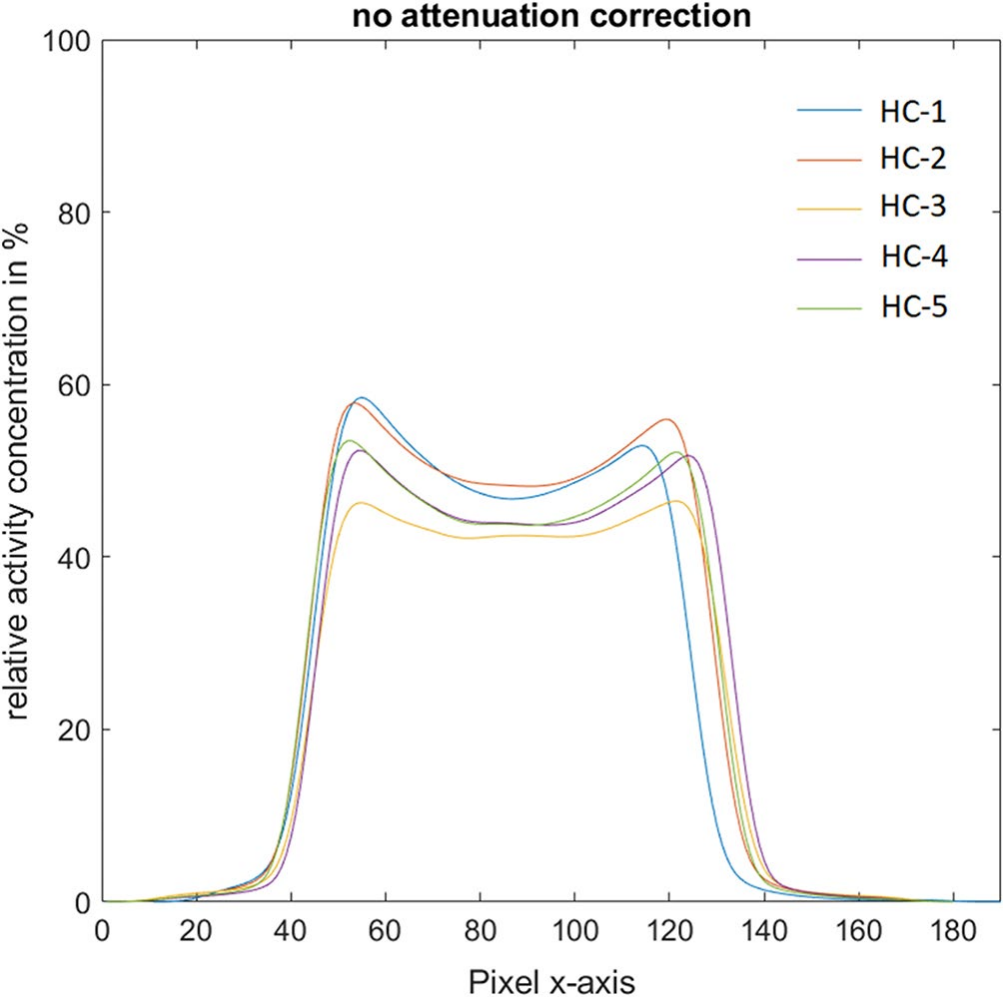
Line profile of all hardware configurations without any attenuation correction showing the measured activity concentration as percentages of the actual activity concentration along the x-axis

Without any hardware parts within the FOV (configuration HC-1), the self-attenuation of the egg phantom caused an underestimation of the activity by an average of 49%. The underestimation was more pronounced in the center of the egg than near the eggshell (Table 1; Fig. 3). Adding the animal cradle (HC-2) and subsequently also the different MRI coils to the configuration (HC-3 to HC-5) caused further, hardware-dependent underestimations (Table 1; Fig. 3).

**Table 1:** Activity concentrations for the different hardware configurations are given for the start of the measurement, respectively. Average activity concentrations were measured in the total egg, the center, and near the eggshell, and calculated as the relative deviation of the actual activity concentration of each configuration in percentages

Hardware configuration	Activity concentration (kBq/mL)	Measured activity total egg (kBq/mL)	Deviation (%)	Measured activity center (kBq/mL)	Deviation center (%)	Measured activity eggshell (kBq/mL)	Deviation eggshell (%)
HC-1	117.0	59.8	-48.8	51.9	-55.7	69.9	-40.2
HC-2	92.6	47.4	-48.9	43.3	-53.3	52.8	-43.0
HC-3	81.6	35.7	-56.2	33.0	-59.6	37.5	-54.1
HC-4	71.0	34.7	-51.2	29.8	-58.1	36.1	-49.2
HC-5	61.4	30.1	-51.0	25.6	-58.4	31.1	-49.4

**Fig. 4:**
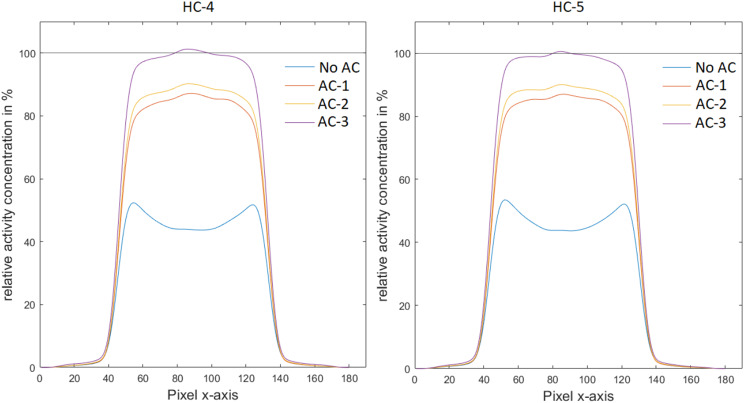
Line profiles through the short axis of the egg phantom for HC-4 and HC-5 showing the effect of the different AC configurations in % deviation of the actual activity concentration. AC included attenuation information (AC-1) for the egg only, (AC-2) for egg and the cradle and (AC-3) for egg cradle and coil

**Fig. 5:**
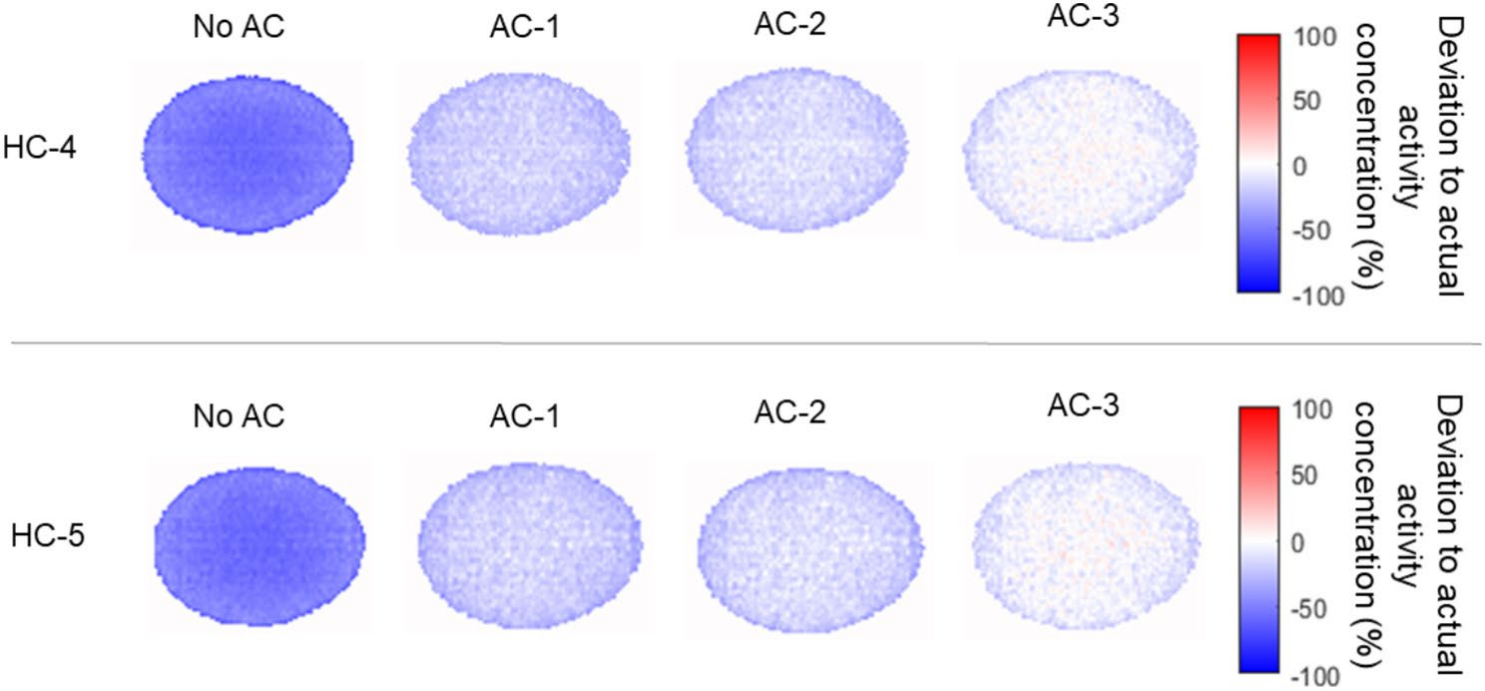
Percent difference images for HC-4 and HC-5 using the different AC configurations. AC included attenuation information (AC-1) for the egg only, (AC-2) for egg and the cradle and (AC-3) for egg cradle and coil

**Fig. 6:**
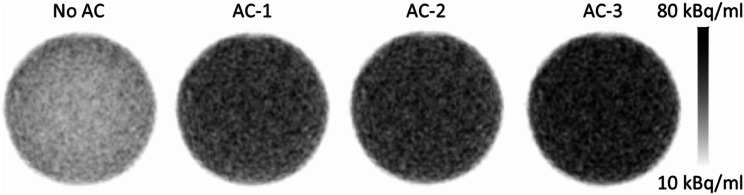
Axial views through the center of one measurement of the egg phantom imaged with HC-5 and reconstructed with the different AC configurations

The general underestimation of activity could be mitigated by including the individual attenuating materials in the AC. Adding only AC for the egg itself (AC-1) reduced the general underestimation of the activity to ~ 20% (Table 2). The behavior was similar for both investigated hardware configurations. Interestingly, the underestimation in the center of the egg was less than that towards the eggshell (Table 2; Fig. 4). Adding the animal bed to the AC further reduced the underestimation of activity and when accounting for all hardware parts in the AC, underestimations were reduced to around 2% for the whole egg with a slight remaining bias gradient between the center of the egg and its edges (Table 2; Fig. 4). Figure 5 depicts the local deviations of activity concentration for all AC implementations. Figure 6 shows an example of the reconstructed PET images for HC-5 for the different AC implementations.

**Table 2:** Average activity concentration in the phantom for HC-4 and HC-5 after using different AC configurations during the PET reconstruction. Measurements for the whole egg, from the center and from near the eggshell are included and relative deviations to the actual activity concentration are given

		Actual activity (kBq/ml)	Measured Activity total egg (kBq/ml)	Deviation (%)	Measured activity center (kBq/ml)	Deviation center (%)	Measured activity eggshell (kBq/ml)	Deviation eggshell (%)
HC-4	no AC	71.0	34.7	-51.2	29.8	-58.1	36.1	-49.2
	AC-1	71.0	57.4	-19.2	60.8	-14.4	56.1	-21.0
	AC-2	71.0	59.5	-16.2	63.4	-10.7	59.5	-16.3
	AC-3	71.0	69.7	-1.9	71.4	0.5	67.4	-5.1
HC-5	no AC	61.4	30.1	-51.0	25.6	-58.4	31.1	-49.4
	AC-1	61.4	49.0	-20.2	52.5	-14.6	50.2	-18.3
	AC-2	61.4	51.1	-16.8	54.7	-11.0	53.2	-13.4
	AC-3	61.4	60.1	-2.2	61.2	-0.4	59.7	-2.8

### *In ovo* PET/MRI

**Fig. 7:**
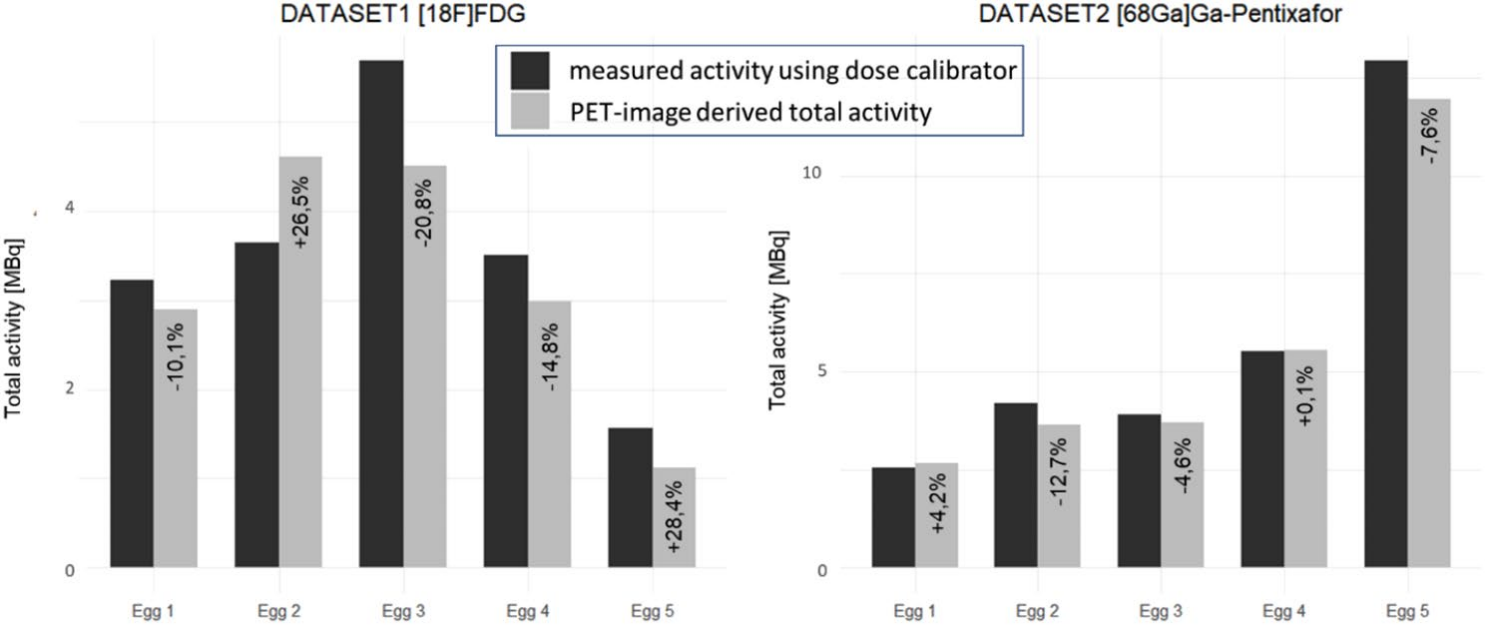
Comparison of the total dose as measured by the dose calibrator and the total dose as assessed from the PET images reconstructed using AC-3

**Fig. 8:**
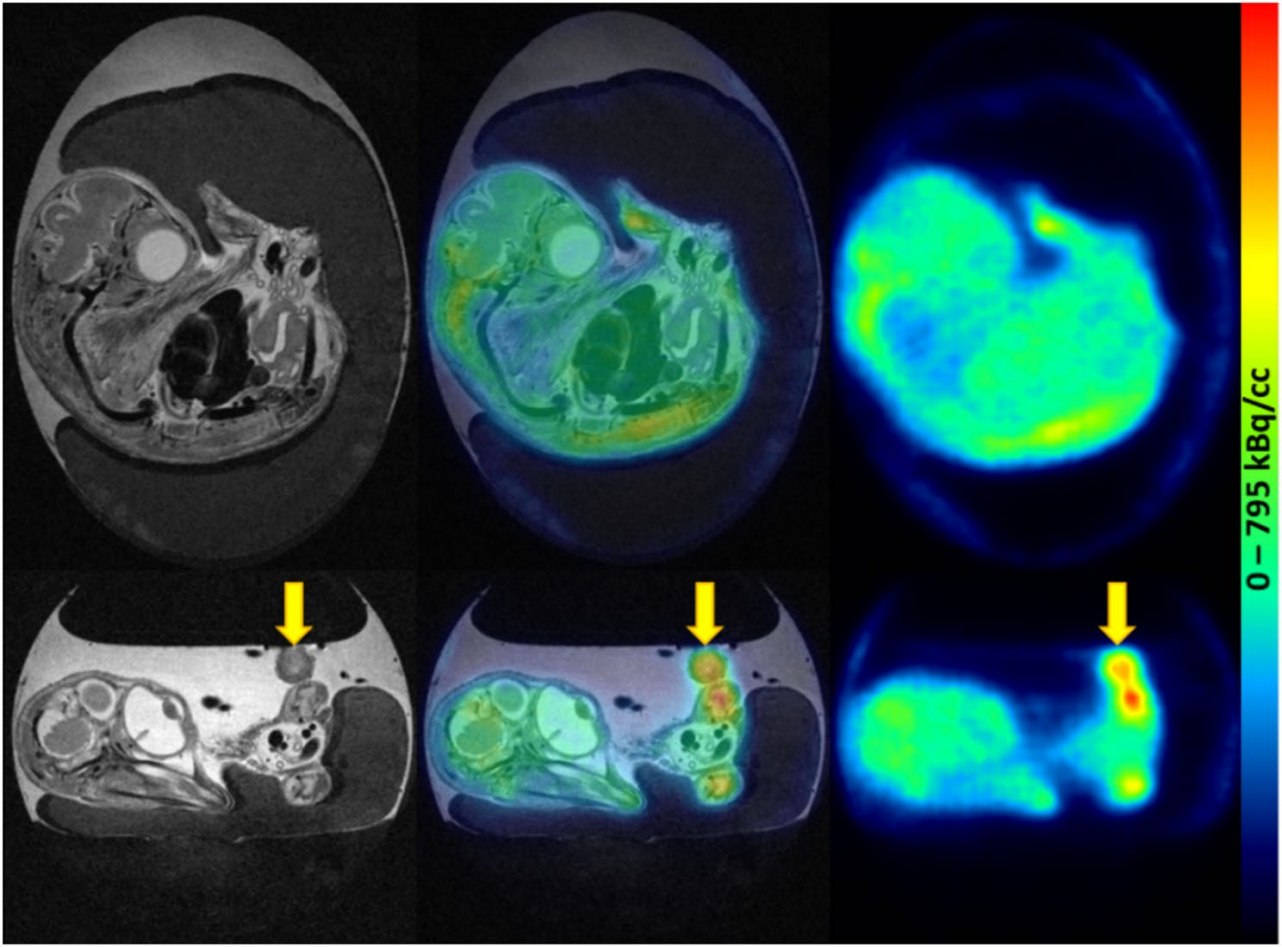
Representative images of [^18^F]FDG distribution in the fertilized chicken egg on EDD18. PET scan was started 60 min p.i. of 5.31 MBq [^18^F]FDG. Left: T2-weighted TurboRARE images, right: PET images, middle: fused PET/MR images. The xenograft (HT29) is marked with yellow arrows

**Fig. 9:**
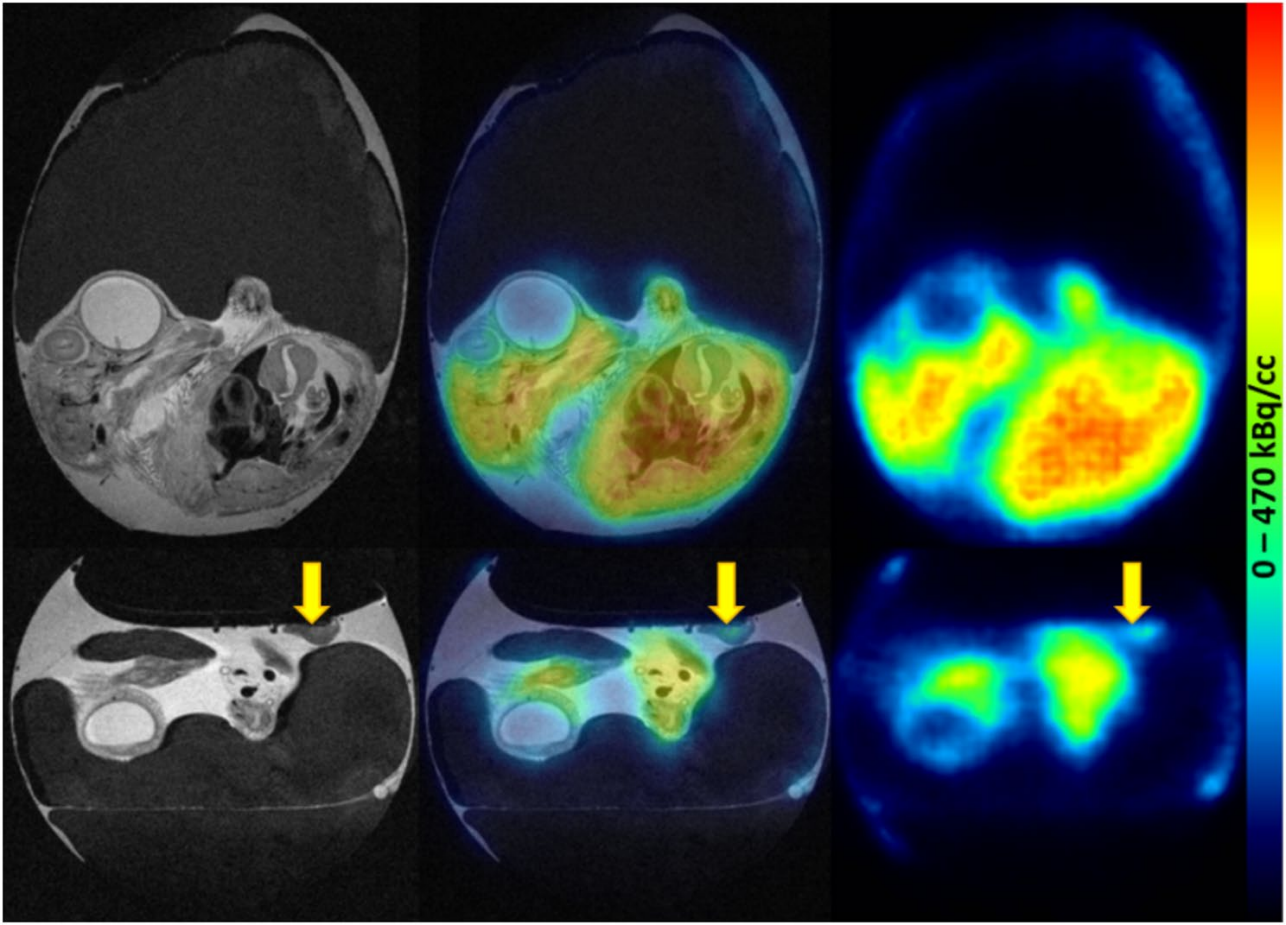
Representative images of [^68^Ga]Ga-Pentixafor distribution in the fertilized chicken egg on EDD16. PET scan was started 60 min p.i. of 7.19 MBq [^68^Ga]Ga-Pentixafor. Left: T2-weighted TurboRARE images, right: PET images, middle: fused PET/MR images. The xenograft (HCT116) is marked with yellow arrows

The measured total activity in the eggs in dataset 1 deferred by -45 ± 10%, -23 ± 16%, -19 ± 17%, and − 10 ± 19% for No AC, AC-1, AC-2, and AC-3, respectively. For dataset 2, deviations of -42 ± 3%, -18 ± 5%, -14 ± 5%, and − 4 ± 6% were found. Interestingly, the variability of the differences was substantially higher for dataset 1, originating from the establishment phase of the methodology (Fig. 7). Two examples of the in ovo experiments can be found in Figs. 8 and 9.

**Fig. 10:**
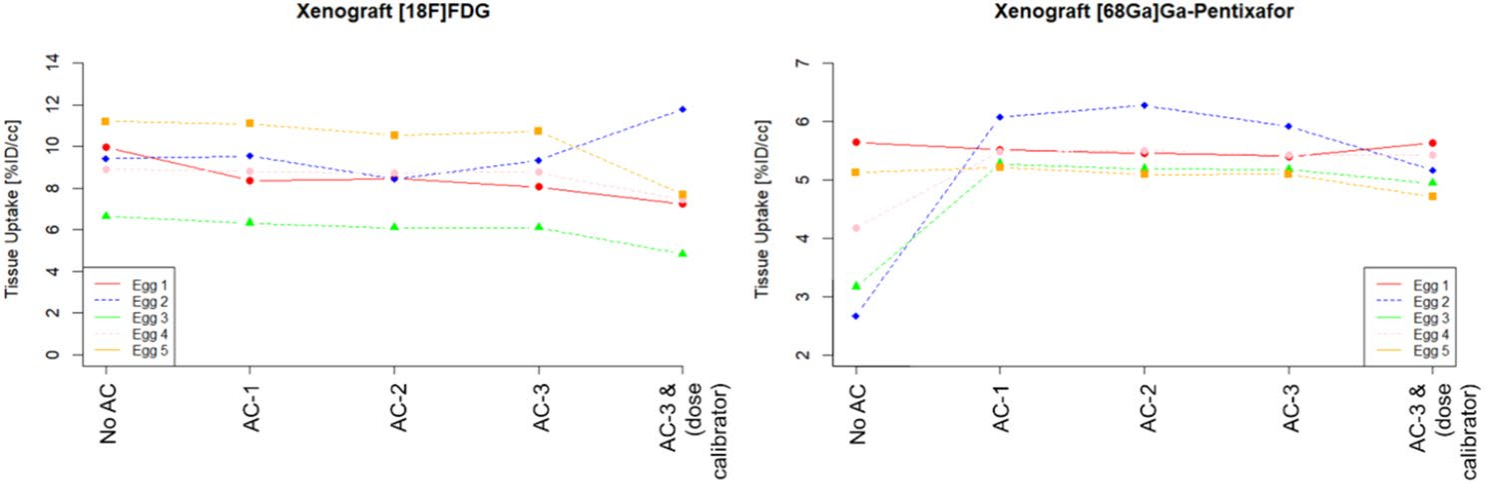
%ID/cc as determined using data from PET following the different AC methods and for AC-3 in addition by using the documented injected dose for normalization

**Fig. 11:**
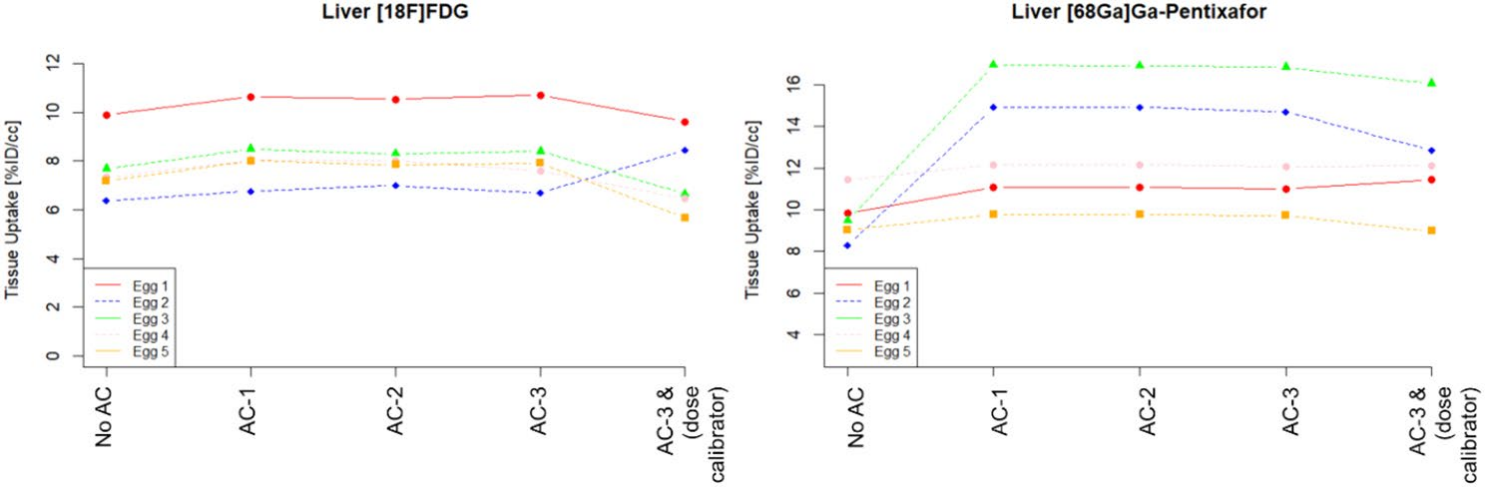
%ID/cc as determined using data from PET following the different AC methods and for AC-3 in addition by using the documented injected dose for normalization

**Fig. 12:**
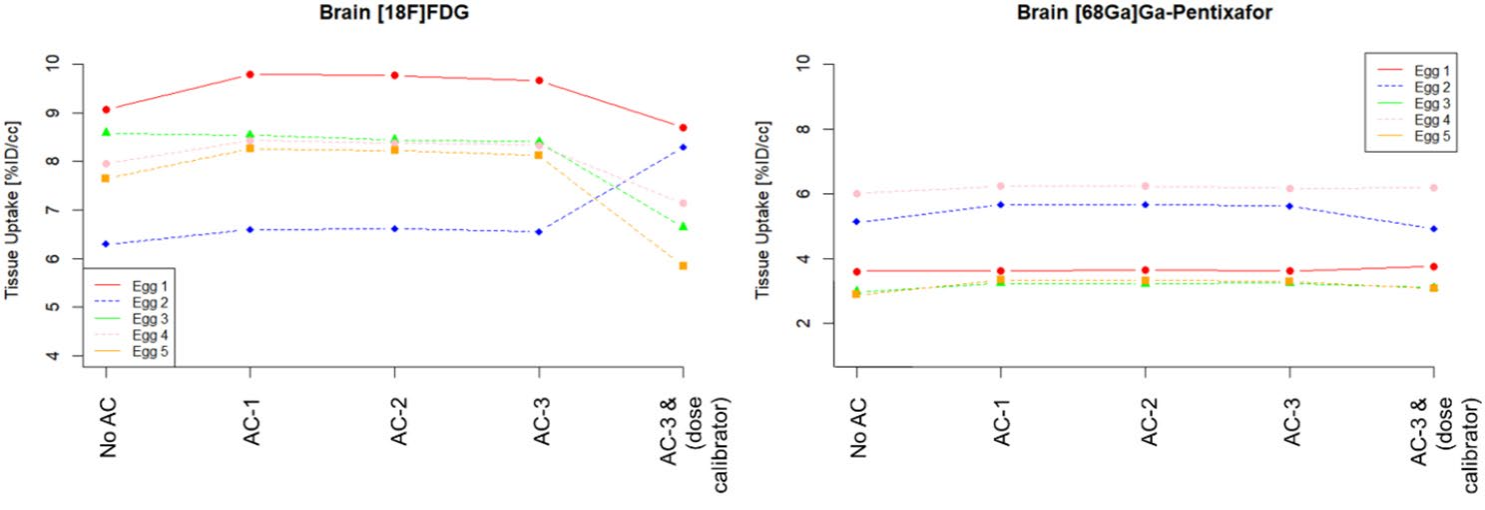
%ID/cc for the chick embryonal brain as determined using data from PET following the different AC methods and for AC-3 in addition by using the documented injected dose for normalization

In general, the quantification based on %ID/cc was similar for different AC configurations (Figs. 10, 11 and 12) when using the image-derived total activity to calculate %ID/cc, with average absolute deviations for both datasets between 1% and 3% of AC-1 and AC-2 compared to AC-3. However, substantial differences to these quantifications were found for %ID/cc calculated from No AC data. This was in particular evident in individual eggs for quantifications of the liver and the xenograft (Figs. 10 and 11). For the quantification of AC-3 data, where %ID/cc was calculated by normalization to the injected activity measured with the dose calibrator, the deviations of %ID/cc to the purely image-based quantification showed the same difference as the quantification of the total activity (Fig. 7).

## Discussion

In this study, the influence of the different sources of attenuation on PET for *in ovo* PET/MRI was evaluated and the accuracy of MR-based AC for *in ovo* PET experiments was assessed. It has been shown that in standard settings including MRI coils and an animal bed, underestimations of activity between 60% and 50% occur in the center and at the edge of the egg, respectively. The quantitative gradient between the center of the egg and its shell is expected and can be explained by the higher self-attenuation for coincidences originating from the inside of the egg. In general, our data shows that the largest contribution to attenuation in *in ovo* studies is the self-attenuation of the egg. This self-attenuation can be effectively compensated by incorporating a simple attenuation approach based on thresholding the egg on MRI and assigning respective attenuation values. In this study, this was done following the standard approach also used for rodents where a linear attenuation coefficient of 0.1023/cm is assumed. As this linear attenuation coefficient is slightly higher than that of water, which was used to fill our phantom, a slight overestimation of the activity concentration might have occurred. In addition, we observed a higher activity concentration in the center of the eggs which we relate to imprecisions from the scatter correction (Fig. 4). Nevertheless, this method enabled a simple compensation of the self-attenuation with practically acceptable results (Table 2). Of note, in this study a physiolocial saline solution was used in the phantom as a surrogate for the different material types within the egg (egg yolk, albumin, the embryonal tissues). However, given the similarties in radiological densities of the different materials and water [23, 24] the results are expacted to represent a realistic estimate of the attenuation effects in in ovo PET. Furthermore, the phantom experiments where performed using fluorine-18 as radionuclide only. However, the results are transferable to any PET nuclide as the filling was homogeneous and thus, positron range effects in individual voxels cancel each other out except for voxels located at the edge of the phantom.

The hardware parts, such as the animal bed and the PET-compatible MRI coils, which are usually present in a preclinical PET/MRI system, caused an overall underestimation of approximately 20% if not considered in the AC (Table 2). This is reasonable given that the animal bed and MRI coils are optimized for the use with the PET insert, and thus, are designed to add only a minimal amount of additional material in the PET FOV. However, the contribution of such hardware parts is substantially higher in the case of non-optimized hardware (see Table 1, results for HC-3). In general, the hardware parts’ contribution to attenuation resulted rather in a global bias within the egg than in local quantification variations (Fig. 4). This was particularly evident for the MRI coils. Therefore, a bias caused by cylindrical MRI coils might be also addressed by a general scaling factor in case a respective attenuation template is not available.

For the in ovo studies, a similar global behavior as observed for the phantoms was found with comparable underestimations of the activities when not accounting for attenuation.

Interestingly, the comparison of the injected activities with the image-derived activities showed substantial deviations for individual eggs of dataset 1 (Fig. 7). This dataset contained eggs from a test run during the establishment phase of the methodology on-site. Troubleshooting revealed that bleeding of CAM vessels regularly occurred after tracer injection during this period. Any radioactive blood residues were dabbed off, and thus, removed from the egg but were partly not measured when determining the residual activity in the syringe after tracer injection (which is normally subtracted from the injected dose). This practical pitfall, which was recently also described elsewhere [11], could be mitigated by injection training to minimize bleeding and a standardized procedure to determine residual activities including eventual activities in used tips. The result of this mitigation strategy was an improvement in accuracy which can be nicely seen in the quantitative assessment of dataset 2 (Fig. 7).

However, a strategy that has proven successful in practice to avoid possible inaccuracies in the measurement of injected activity is to use the total activity in the egg, determined from the PET images, to calculate %ID/cc. This is possible as chicken eggs have dimensions of ~ 5 cm which are fully covered by preclinical PET FOVs [25]. As long as the self-attenuation of the egg was corrected, this method provided quantitative results within a spread of a few percent even when ignoring the hardware parts of the MRI. Only, if not accounting for the self-attenuation of the egg, a substantial variability of the quantitative accuracy depending on the location of the target tissue within the egg was found. A good example for such variabilities can be seen in the xenograft or liver uptake of the [^68^Ga]Ga-Pentixafor experiments (Figs. 10 and 11). Such behaviour is expected due to the location dependency of the influence of the self attenuation and compares well with the findings in the phantom.

Limitations: This study investigated the influence of attenuation on the quantification of in ovo PET. Nevertheless, other factors such as scatter radiation and respective correction or the use of different reconstruction algorithms and point spread function correction also have an influence on quantification. However, in this preclinical system, scatter estimation and correction is based on the dual energy window method. This means that the scatter contribution is solely estimated based on the PET emission data, and thus, in contrast to clinical systems where scatter correction is usually based on a single scatter simulation, not connected to the AC method. Therefore, the results in this study should be independent from scatter correction.

In this study, the actual activity concentration in the embryo organs and xenografts was not measured. The use of different reconstruction setting such as iterations and subsets, post reconstruction fitlers or PSF correction can influence the appearance and quantification of small structures [26, 27]. These parameters have an influence on the spatial resolution and the convergence of small objects in iterative reconstructions. The effects are expected to have a substantial influence in e.g. the xenografts when extracting the true activities or measurements of the signal to background ratio and should be considered when comparing PET measurements with published literature or biodistribution measurements based on gamma counters. However, with regards to this study, AC is a multiplicative factor within the reconstruction and should not have an influence on local convergence or resolution of the PET images irrespective of the used reconstruction algorithm [28]. Therefore, the results of this study regarding the bias introduced by the different AC strategies are expected to be directly applicable to other reconstruction settings such as including PSF correction.

## Conclusion

Self-attenuation of the egg and PET signal attenuation within the hardware parts of the MRI substantially influence quantitative accuracy in *in ovo* measurements. However, as long as the self-attenuation of the egg is compensated through a respective attenuation correction, a reliable quantification by means of %ID/cc can be performed by using the total activity within the egg measured in the image data as injected dose. This method also compensates for practical and experimental pitfalls in the assessment of the injected dose using a dose calibrator.

## Data Availability

The datasets generated and analysed during the current study are available from the corresponding author on reasonable request.
